# Interrelations between migraine-like headache and persistent post-traumatic headache attributed to mild traumatic brain injury: a prospective diary study

**DOI:** 10.1186/s10194-020-01202-6

**Published:** 2020-11-19

**Authors:** Håkan Ashina, Afrim Iljazi, Faisal M. Amin, Messoud Ashina, Richard B. Lipton, Henrik W. Schytz

**Affiliations:** 1 Danish Headache Center, Department of Neurology, Rigshospitalet Glostrup, Faculty of Health and Medical Sciences, University of Copenhagen, Valdemar Hansen Vej 5, Glostrup, DK-2600 Copenhagen, Denmark; 2 Department of Neurology, Albert Einstein College of Medicine, Bronx, NY USA; 3 Montefiore Medical Center, Bronx, NY USA

**Keywords:** Concussion, Head trauma, Diagnosis, Clinical characteristics

## Abstract

**Background:**

Persistent post-traumatic headache (PTH) is a common sequela of mild traumatic brain injury (TBI) and retrospective assessments have found a migraine-like phenotype to be very frequent. This has raised a discussion of shared underlying mechanisms and whether persistent PTH is simply trauma-triggered migraine.

**Methods:**

A 28-day prospective diary study with daily entries and acquisition of data on headache characteristics, associated symptoms, and acute medication use. A total of 64 patients with persistent PTH were enrolled from April 2019 to August 2019. Outcomes were the proportion of monthly headache days of any intensity that met the criteria for a migraine-like day or TTH-like day, as well as the corresponding figures for monthly headache days of moderate to severe intensity. Headache phenotypes were initially assigned based on diagnostic evaluation by semi-structured interview, whilst final headache phenotypes were assigned by diary review.

**Results:**

After diary review, we found that monthly headache days were exclusively migraine-like in 24 of 64 patients (38%) and exclusively TTH-like days in 8 of 64 patients (13%). Considering only monthly headache days of moderate to severe intensity, the corresponding figures were 35 of 64 patients (55%) for migraine-like days and 8 of 64 patients (13%) for TTH-like days. The following headache phenotypes were assigned based on diary review: chronic migraine-like (*n* = 47, 73%), combined episodic migraine-like and chronic TTH-like (*n* = 9, 13%), and ‘pure’ chronic TTH-like (*n* = 8, 13%).

**Conclusions:**

A migraine-like phenotype is common in patients most adversely affected by persistent PTH, although some patients did have a pure chronic TTH-like phenotype. At minimum, these findings suggest that persistent PTH is – at least in some – not ‘trauma-triggered migraine’.

## Introduction

Post-traumatic headache (PTH) is a disabling headache disorder and a common sequela of mild traumatic brain injury (TBI), [1]. The Third Edition of the International Classification of Headache Disorders (ICHD-3) defines PTH as a secondary headache disorder attributed to either mild TBI or moderate to severe TBI [2]. PTH is characterized by onset of headache within 7 days of mild TBI and, if the headache persists for at least 3 months, the term persistent PTH is used [2].

While ICHD-3 conceptualizes persistent PTH as a secondary headache disorder, the definition and diagnosis remains controversial and some contest that persistent PTH is ‘trauma-triggered migraine [3]. This is supported by the observation that persistent PTH most often mimics a migraine-like headache phenotype in clinic-based studies [4, 5], leading to use of the term ‘post-traumatic migraine’ [6]. Current therapeutic strategies for persistent PTH includes acute and preventive medications used for the treatment of migraine [4, 7], with only some supportive evidence of this approach from open-label studies in individuals with persistent PTH [8, 9]. It could be speculated that persistent PTH sometimes represents migraine unmasked by mild TBI [10].

As a step towards clarifying the relationship of persistent PTH to migraine, we performed a 28-day prospective diary study in patients with persistent PTH who had at least eight monthly headache days of moderate to severe intensity. Daily diary entries were used to classify each headache day by the phenotype it most mimics. Furthermore, we assigned a final headache phenotype diagnosis based on diary review, without consideration of the ICHD-3 criterion for migraine and tension-type headache (TTH) which states “not better accounted for by another ICHD-3 diagnosis” [2]. The aim was to characterize the range of headache phenotypes in individuals with persistent PTH and shed light on the similarities and differences between persistent PTH and migraine.

## Methods

### Overview

This study was approved by the Regional Health Research Ethics Committee of the Capital Region of Denmark (identifier: H-18050498), the Danish Medicines Agency (identifier: 2018–1104), and the Danish Data Protection Agency (identifier: VD-2019-20). Written informed consent was obtained from each participant before any study procedures or assessments were performed. The study was conducted in accordance with the Declaration of Helsinki [11].

Patients were recruited from an open-label trial set at the Danish Headache Center, Rigshospitalet Glostrup, Denmark [8]. Enrollment took place from April 2019 to August 2019. At the screening visit, site investigators performed an in-person semi-structured interview to record data on demographics, medical history, and full clinical course [8]. Patients were then enrolled in a 28-day paper diary study with daily entries and trained by the site investigators on completion of their diary.

Eligible patients included men or women aged 18 to 65 years with personal history of persistent headache attributed to mild TBI, in accordance with the ICHD-3 [2]. While ICHD-3 criteria defines persistent PTH by duration of headache for at least 3 months after mild TBI, we only included patients who had a history of persistent PTH for more than 12 months after mild TBI. Eligible patients were also required to report at least eight monthly headache days of moderate to severe intensity at the screening visit. Exclusion criteria for the diary study included *1)* any personal history of primary headache disorder, except infrequent TTH pre-trauma, *2)* any personal history of whiplash injury, and *3)* medication-overuse headache, as defined by ICHD-3 [2]. To further ensure study eligibility, medical records were reviewed to cross-check for any formal headache diagnosis code. The full list of exclusion criteria has been published elsewhere [8].

### Measures and definitions

A 28-day diagnostic headache diary was used to prospectively collect information on headache characteristics, associated symptoms, and use of acute medication. Patients were instructed to answer a daily set of questions at the end of each day and had been informed at the screening visit that the recall period was defined as the past 24 h.

- A headache day of moderate to severe intensity was defined as any day with headache of moderate to severe intensity that lasted at least 4 h.
- A migraine-like day was defined as any day with headache that lasted at least 4 h and fulfilled the ICHD-3 criteria for migraine without aura, migraine with aura, or probable migraine (2). Of note, patients were assigned with a TTH-like day if they met the criteria for probable migraine but also those for TTH. Furthermore, we also considered a migraine-like day to be defined by successful response to acute treatment with migraine-specific medication (i.e. triptan, ditan, gepant, ergot alkaloid).
- A TTH-like day was defined as any day with headache that lasted at least 30 min and fulfilled the ICHD-3 criteria for TTH [2].

Headache phenotypes were initially assigned based on diagnostic evaluation by trained medical students, while final headache phenotypes were assigned after diary review by two headache specialists (M.A. and H.W.S). Possible headache phenotypes included the following options:

- *Episodic migraine-like*, defined as headache occurring on ≥1 day per month, which exclusively fulfills the criteria for a migraine-like day, but does not meet the requirement for a chronic migraine-like phenotype (see below).
- *Chronic migraine-like*, defined as headache occurring on ≥15 days per month, which, on ≥8 days per month fulfill the criteria for a migraine-like day.
- *Episodic TTH-like*, defined as headache occurring on 1–14 days per month, which exclusively fulfills the criteria for a TTH-like day.
- *Chronic TTH-like*, defined as headache occurring on ≥15 days per month, which exclusively fulfills the criteria for a TTH-like day.
- *Combined episodic migraine-like and episodic TTH-like*, defined as headache occurring on 2–15 days per month, which, on ≥1 day per month fulfills the criteria for a migraine-like day, and on ≥1 day per month fulfills the criteria for a TTH-like day, but does not meet the requirement for a chronic migraine-like phenotype (see above).
- *Combined episodic migraine-like and chronic TTH-like*, defined as headache occurring on > 15 days per month, which, on ≥15 days per months fulfills the criteria for a TTH-like day, and on 1–7 days per months fulfills the criteria for a migraine-like day.

### Statistical analysis

Outcomes were calculated based on headache diary entries for all patients who demonstrated at least 90% compliance throughout the 28-day diary period. For each patient, we calculated the following: 1) number of monthly headache days of any intensity, 2) number of monthly headache days of moderate to severe intensity, 3) proportion of monthly headache days of any intensity that met the criteria for a migraine-like day or a TTH-like day, and 4) proportion of monthly headache of moderate to severe intensity that met the criteria for a migraine-like day or a TTH-like day. In addition, we examined the proportion of patients whose monthly headache days of any intensity were 100% migraine-like, ≥75% migraine-like, 100% TTH-like, or ≥ 75% TTH-like. The latter calculations were also performed separately for monthly headache days of moderate to severe intensity. R statistical software version 3.6.0 was used to generate all data listing, summaries, and statistical analyses.

## Results

A total of 64 patients with persistent PTH were included in this 4-week prospective diary study. The mean (SD) age was 34.0 (11.1) years, and the mean (SD) years of education was 14.8 (2.9). Of 64 patients, 49 (77%) were females and 15 (23%) were males. The mean (SD) number of months with headache attributed to mild TBI was 61.3 (60.0). Characteristics of the study population are summarized in Table 1. The mean (SD) headache frequency was 26.3 (2.9) days per month, while the mean (SD) headache frequency of moderate to severe intensity was 19.7 (6.6) days per month. A positive family history of migraine was reported by 32% of patients. In terms of treatment patterns, 58 of 64 patients (91%) reported current use of acute medication, with 30 patients (47%) having current or previous use of triptans. Of the latter, 17 of 30 patients (57%) reported insufficient pain relief. A history of preventive medication use was reported by 49 patients, of whom 27 (55%) reported failure of at least two preventive medications. Current use of preventive medication was reported by 29 patients (45%).

**Table 1:** Characteristics of the Study Population

Variable	Persistent PTH (*n* = 64)
**Age**, mean (SD), y	34.0 (11.1)
**Male/Female**, %	23/77
**Body Mass Index,** mean (SD), kg/m^2^	26.2 (5.6)
**Current Employment Status**, %	
Full-time employed	22
Part-time employed	53
Unemployed	22
Retired	3
**Education**	
Years of education, mean (SD), y	14.8 (2.9)
Level of education, %	
No completed education besides secondary school or high school	17
Skilled labor	28
Bachelor’s degree	27
Higher education	28
**Injury Cause**, %	
Fall	28
Motor vehicle collision	27
Sports-related injury	20
Violence/assault	5
Other unintentional injury	20
**Months with Headache attributed to Mild TBI**, mean (SD), months	61.3 (60.0)
**Family History of Migraine**, %	32
**Headache Frequency**, mean (SD)	
Monthly headache days of any intensity	26.3 (2.9)
Monthly headache days of moderate to severe intensity	19.7 (6.6)
**Acute Medication Use**	
**Current Acute Medication Use**, No. of subjects (%)	58 (91)
**Triptans**	
Current or previous use, No. of subjects (%)	30 (47)
Lack of efficacy, No. of subjects (%)	17 (57)
**Prevention Medication Use**	
**History of Preventive Medication Use**, No. of subjects	49
No drug failures, %	14
Failure of ≥1 drug, %	86
Failure of ≥2 drugs, %	55
Failure of ≥3 drugs, %	37
Failure of ≥4 drugs, %	20
**Treatment Naïve**, No. of subjects	15
**Current Preventive Medication Use**, No. of subjects	29

Two headache specialists (M.A. and H.W.S.) assigned final headache phenotype diagnosis based on review of the prospectively collected 28-day headache diary entries (κ = 1). The most common phenotype was chronic migraine-like (*n* = 47, 73%) followed by a combined episodic migraine-like and chronic TTH-like (*n* = 9, 14%) and pure chronic TTH-like (*n* = 8, 13%), (Table 2). The mean (SD) headache frequency was 26.6 (2.4) days per month in patients with a chronic migraine-like phenotype, 24.6 (3.8) days per month in those with a combined episodic migraine-like and chronic TTH-like phenotype, and 26.6 (3.6) days per month in patients with a pure chronic TTH-like phenotype. In terms of headache days of moderate to severe intensity, the mean (SD) frequency was 19.9 (6.5) days per month in patients with a chronic migraine-like phenotype, 17.7 (7.0) days per month in those with a combined episodic migraine-like and chronic TTH-like phenotype, and 20.3 (5.9) days per month in patients with a pure chronic TTH-like phenotype. A positive family history of migraine was reported by 32% with a chronic migraine-like phenotype (*n =* 47), 44% with a combined episodic migraine-like and chronic TTH-like phenotype (*n =* 9), and 38% with a ‘pure’ chronic TTH-like phenotype (*n =* 8).

**Table 2:** Characteristics of Study Population based on Headache Phenotype Diagnosis following Diary Review

Variable	Chronic Migraine-Like (*n* = 47)	Episodic Migraine-Like combined with TTH-Like (*n* = 9)	Chronic TTH-Like (*n* = 8)
**Age**, mean (SD), y	34.5 (12.0)	34.3 (7.9)	31.5 (8.0)
**Male/Female**, %	19/81	33/67	37/63
**Body Mass Index,** mean (SD), kg/m^2^	26.1 (5.6)	24.6 (3.8)	24.9 (4.0)
**Current Employment Status**, No. of subjects (%)			
Full-time employed	10 (21)	2 (22)	2 (25)
Part-time employed	25 (53)	4 (44)	5 (63)
Unemployed	11 (23)	2 (22)	1 (13)
Retired	1 (2)	1 (11)	0 (0)
**Education**			
Years of education, mean (SD), y	14.3 (2.6)	16.3 (3.2)	15.9 (3.3)
Level of education, No. of subjects (%)			
Secondary school or high school	10 (21)	0 (0)	1 (13)
Skilled labor	13 (28)	4 (44)	1 (13)
Bachelor’s degree	11 (23)	3 (33)	3 (38)
Higher education	13 (28)	2 (22)	3 (38)
**Injury Cause**, No. of subjects, %			
Fall	12 (26)	3 (33)	3 (38)
Motor vehicle collision	14 (30)	2 (22)	1 (13)
Sports-related injury	11 (23)	1 (11)	1 (13)
Violence/assault	1 (2)	1 (11)	1 (13)
Other unintentional injury	9 (19)	2 (22)	2 (25)
**Months with Headache attributed to Mild TBI**, mean (SD), months	55.0 (55.1)	89.0 (73.4)	70.0 (58.0)
**Family History of Migraine**, No. of subjects (%)	15 (32)	4 (44)	3 (38)
**Headache Frequency**, mean (SD)			
Monthly headache days of any intensity	26.6 (2.4)	24.6 (3.8)	26.6 (3.6)
Monthly headache days of moderate to severe intensity	19.9 (6.5)	17.7 (7.0)	20.3 (5.9)
**Migraine-Like Frequency**, mean (SD)			
Monthly migraine-like days	22.5 (6.8)	3.6 (1.9)	–
Monthly definite migraine-like days	17.6 (8.4)	3.0 (2.1)	–
Monthly probable migraine-like days	4.8 (6.1)	0.6 (1.1)	–
**TTH-Like Frequency**, mean (SD)			
Monthly TTH-like days	4.1 (6.3)	21.0 (4.6)	26.6 (3.6)
**Acute Medication Use**			
**Current Acute Medication Use**, No. of subjects (%)	44 (94)	7 (78)	7 (88)
**Triptans**			
Current or previous use, No. of subjects	24	5	1
Lack of efficacy in those with history of acute medication use, No. of subjects (%)	13 (54)	3 (60)	1 (100)
**Prevention Medication Use**			
**History of Preventive Medication Use**, No. of subjects (%)	36 (77)	7 (78)	6 (75)
No drug failures, No. of subjects (%)	4 (11)	2 (29)	1 (17)
Failure of ≥2 drugs, No. of subjects (%)	20 (56)	5 (71)	2 (33)
**Preventive Treatment Naïve**, No. of subjects	11 (23)	2 (22)	2 (25)
**Current Preventive Medication Use**, No. of subjects (%)	20 (43)	5 (56)	4 (50)

### Migraine-like days and TTH-like days

After diary review (Table 3), 35 of 64 patients (55%) had at least 75% overlap of monthly migraine-like days with monthly headache days. If considering only overlap of monthly migraine-like days with monthly headache days of moderate to severe intensity, 35 of 64 patients (55%) had 100% overlap while 39 of 64 (61%) at least 75% overlap.

**Table 3:** Summary of Study Outcomes

	Persistent PTH (*n* = 64)
**Headache Frequency during the 4-Week Diary Period**, mean (SD)	
Headache days of any intensity	26.3 (2.9)
Headache days of moderate to severe intensity	19.7 (6.6)
**Monthly Headache Days**, No. of subjects (%)	
Monthly headache days of any intensity were exclusively migraine-like days	24 (38)
At least 75% of monthly headache days of any intensity were migraine-like days	35 (55)
Monthly headache days of any intensity were exclusively TTH-like days	8 (13)
At least 75% of monthly headache days of any intensity were TTH-like days	16 (25)
**Monthly Headache Days of Moderate to Severe Intensity**, No. of subjects (%)	
Monthly headache days of moderate to severe intensity were exclusively migraine-like days	35 (55)
At least 75% of monthly headache days of moderate to severe intensity were migraine-like days	39 (61)
Monthly headache days of moderate to severe intensity were exclusively TTH-like days	8 (13)
At least 75% of monthly headache days of moderate to severe intensity were TTH-like days	15 (23)

**Table 4:** Reclassification of Initial Headache Phenotype Diagnosis based on Diary Review

Initial Headache Phenotype based on Semi-structured Interview, No. of subjects	Final Headache Phenotype based on Diary Review, No. of subjects			Total, No. of subjects (%)
	CM-Like	EM-Like + CTTH-Like	CTTH-Like	
CM-Like	**42**	**2**	**1**	**45 (70)**
EM-Like + CTTH-Like	**2**	**7**	**2**	**11 (17)**
CTTH-Like	**3**	**0**	**5**	**8 (13)**
**Total, No. of subjects (%)**	**47 (73)**	**9 (14)**	**8 (13)**	**64 (100)**

*No.* Number, *CM-Like* Chronic Migraine-Like, *EM-Like* Episodic Migraine-Like, *CTTH-Like* Chronic TTH-Like

Eight of 64 patients (13%) had 100% overlap of monthly TTH-like days with monthly headache days, whereas a corresponding overlap of at least 75% was found in 16 of 64 patients (25%), (Table 3). If considering only overlap of monthly TTH-like days with monthly headache days of moderate to severe intensity, 9 of 64 (13%) had 100% overlap while 15 of 64 (23%) had at least 75% overlap.

### Reclassification of initial headache phenotype diagnosis based on diary review

At the initial diagnostic evaluation, the most common phenotypes assigned were chronic migraine-like (*n* = 45, 70%) followed by combined episodic migraine-like and chronic TTH-like (*n* = 11, 17%), and pure chronic TTH-like (*n* = 8, 13%), (Table 4). After a diary review, the corresponding figures were 73%, 14%, and 13%. Ten of 64 patients had their initial headache phenotype diagnosis reclassified based on diary review (Table 4). Trained medical students were able to provide an accurate initial headache phenotype diagnosis in 84% of patients by retrospective assessment using a semi-structured interview.

## Discussion

In this study of 64 patients with persistent PTH and at least eight monthly headache days of moderate to severe intensity, we collected prospective diary data to examine the overlap of monthly migraine-like days with monthly headache days. Taken together, we observed that most patients were assigned with a chronic migraine-like phenotype and monthly headache days more often overlapped with monthly migraine-like days than monthly TTH-like days. However, we did also find that some patients had 100% overlap between monthly TTH-like days and monthly headache days and were, thus, assigned with a pure chronic TTH-like phenotype. At minimum, our findings establish that not all individuals with persistent PTH have a migraine-like phenotype. It should, furthermore, be emphasized that none of the included patients had a personal history of migraine.

Phenotyping of clinical features are important to establish a more comprehensive and symptom-based classification of PTH [12]. The ICHD-3 recommends phenotyping of a patient’s clinical features using a headache diary when more than one headache type/subtype is suspected [2]. However, phenotyping of acute and persistent PTH has only been made with methods that are limited by recall bias, e.g. questionnaires and interviews [4, 13–17]. The present study provides the first diary data, in which clinical features of persistent PTH were collected daily. Future diary studies should include larger cohorts to identify subgroups of patients with PTH based on their headache phenotype (e.g. migraine-like, TTH-like) and personal history of primary headache disorder (e.g. pre-existing migraine).

A considerable limitation of the current ICHD-3 diagnostic criteria is its emphasis on expert opinion rather than scientific evidence, which is largely attributed to the scarcity of literature on PTH [2, 10, 13]. Currently the ICHD-3 criteria classifies persistent PTH as ‘any headache’, which develops within 7 days after TBI and persists for more than 3 months after its onset [2]. The implications are that clinicians are left without information on clinical features or frequency thresholds. Fortunately, there is an increasing number of observational clinic-based studies being published, in which headache phenotypes have been assigned. These studies have found that the clinical features of PTH often mimics a migraine-like or TTH-like phenotype but were limited by their method of assessment, i.e. questionnaires and interview [4, 13–17]. Nonetheless, their findings were confirmed in our prospective diary study, in which all headache days reported could be classified as either migraine-like days or TTH-like days. It remains unknown whether treatment strategies guided by assigned headache phenotypes holds any value [9]. Nonetheless, it would be interesting to explore this issue further in the future.

7An important implication of the present diary study is that some individuals with persistent PTH do not experience migraine-like headache, which confirms previous reports based on phenotype assessment by questionnaire or interview [4, 13–17]. Thus, it would be erroneous to continue the discussion of whether persistent PTH is trauma-triggered migraine. At the most, it could be speculated that migraine was unmasked by mild TBI in those with a migraine-like phenotype. If so, biological underpinnings should be similar between the two groups. This does not seem to be the case based on findings from magnetic resonance imaging (MRI) studies, in which differences in brain structure and function were found when comparing patients with persistent PTH to patients with migraine [18–21]. It should be mentioned that the included patients with persistent PTH were mostly assigned with a migraine-like phenotype, although they did not have a personal history of migraine [18–21]. These data are suggestive of persistent PTH not being trauma-triggered migraine [10, 21]. Furthermore, one recent study explored plasma levels of calcitonin gene-related peptide (CGRP) in individuals with persistent PTH [22]. The authors found that CGRP plasma levels were lower in 100 individuals with persistent PTH when compared with 100 age- and gender-matched healthy non-headache controls. This observation is interesting given that elevated plasma/serum levels of CGRP have recently been proposed as a biomarker candidate for the ictal phase of migraine [23–25]. It should also be mentioned that numerous randomized clinical trials (RCTs) have demonstrated therapeutic benefit of monoclonal antibodies (mAbs) targeting CGRP or its receptor for migraine prevention [26–33]. Although there is no comparable RCT data, one 12-week open-label trial did find that 28% of individuals with persistent PTH experienced at 50% reduction in monthly headache days of moderate to severe intensity after treatment with a mAb targeting the CGRP receptor (erenumab), [8]. Taken together, more research is needed to ascertain similarities and differences between persistent PTH and migraine [34–38]. Such investigations should also assess the role of family history of migraine, response to migraine-specific medication, as well as involvement of biomarkers associated with migraine (e.g. genetic, provocation, biochemistry, imaging).

### Limitations

A limitation of the present study was the modest sample size and a study population which only included patients with persistent PTH who experienced at least eight monthly headache days of moderate to severe intensity. Thus, our study population is skewed towards reflecting more adversely affected individuals with persistent PTH in the general population. However, this was intended as we aimed to assess the overlap of monthly migraine-like days with monthly headache days in patients most burdened by persistent PTH. Another limitation was that our outcomes were assessed by entries in a paper format diary, which does not allow monitoring of daily entries. However, the included patients had been informed at the screening visit that the recall period was defined as the past 24 h.

## Conclusions

A migraine-like phenotype is most common in patients most adversely affected by persistent PTH, although a substantial minority of patients do have a pure TTH-like phenotype. These findings – at minimum – dismiss the notion that persistent PTH is trauma-triggered migraine. Further studies are needed to ascertain similarities and differences of biological underpinnings between persistent PTH and migraine.

## Data Availability

Qualified researchers can request access to patient-level data and related study documents, including the study protocol. Patient-level data will be deidentified and study documents will be redacted to protect the privacy of study participants.

## Abbreviations

PTH: Post-traumatic headache
TBI: Traumatic brain injury
ICHD-3: International Classification of Headache Disorders, Third Edition
TTH: Tension-type headache
CGRP: Calcitonin gene-related peptide
RCT: Randomized clinical trial
mAbs: Monoclonal antibodies
